# A diabetes peer support intervention: Patient experiences using the Mmogo-method®

**DOI:** 10.4102/hsag.v26i0.1512

**Published:** 2021-01-27

**Authors:** Melanie A. Pienaar, Marianne Reid

**Affiliations:** 1 School of Nursing, Faculty of Health Sciences, University of Free State, Bloemfontein, South Africa

**Keywords:** Mmogo-method®, patient experiences, peer support, South Africa, type 2 diabetes

## Abstract

**Background:**

Self-management is the backbone of diabetes care. For the patient with type 2 diabetes, this implies making decisions about a healthy diet, regular exercise and taking treatment appropriately. Some patients may experience barriers to the self-management of diabetes, such as lack of support. In this respect, peer support has been identified as a promising strategy in the self-management of diabetes.

**Aim:**

The study aimed to explore the experiences of adults with type 2 diabetes who took part in a diabetes peer support intervention in the Free State, South Africa. Such information may lead to the development of practical methods for diabetes self-management and control.

**Methods:**

Twelve purposively sampled Sesotho-speaking women (aged 51–84 years) participated in the Mmogo-method®, a visual-based narrative enquiry. Textual data from audio recordings of discussions, visual data from photographs of constructions and field notes were triangulated and analysed thematically.

**Results:**

Participants described the peer support intervention as very valuable. They regarded community health workers as an important source of support. Three themes emerged from the intervention: positive lifestyle changes, continuous support, and improved confidence and sense of connectedness. This was a significant finding reported in patients with diabetes, as it will contribute to successfully sustaining effective self-management behaviour.

**Conclusions:**

Peer support for patients with type 2 diabetes appeared to be a valued intervention, as participants related well to community health workers, who are ideally positioned in the healthcare system to provide the service.

## Introduction

Diabetes is a global health pandemic that currently affects more than 463 million people. It is expected that by the year 2045, 700 million people will be living with diabetes (International Diabetes Federation 2019). As self-management is the basis of diabetes control (Wu, Tai & Sun 2019), new effective ways are needed to improve the self-management of diabetes. The World Health Organisation has identified peer support as a promising approach, which may assist in the self-management of diabetes (World Health Organization 2007).

Peer support refers to:

[*T*]he provision of emotional, appraisal, and informational assistance by a created social network member who possesses experiential knowledge of a specific behaviour or stressor and similar characteristics as the target population, to address a health-related issue of a potentially or actually stressed focal person. (Dennis 2003:329)

The key components of peer support include *emotional* support, *informational* support, *appraisal* support and *instrumental* support (De Vries et al. 2014; Egbujie et al. 2018). Emotional support may be associated with the sharing of lived experiences (Heisler et al. 2010; Ju et al. 2018), needing a ‘shoulder to cry on’ and/or simply someone to talk to and may be associated with building a relationship of trust (Yeung et al. 2018). Informational support relates to sharing information and assimilating new knowledge and skills (Krishnamoorthy et al. 2018; Yeung et al. 2018), and appraisal support refers to affirming feelings and behaviour (Dennis 2003). Instrumental support may relate to linking individuals with resources in the community and with healthcare professionals (Urichuk et al. 2018). There is a large body of evidence that supports the positive effects of peer support (Qi et al. 2015; Debussche et al. 2018), although some authors are concerned about the inconsistency of findings (Smith et al. 2011; Dale et al. 2012). However, little is known about the experiences of the patients who took part in diabetic peer support interventions.

The aim of this study was to explore the experiences of adults with type 2 diabetes who took part in a diabetes peer support intervention that used the Mmogo-method®. Such information may lead to the development of practical methods of diabetes self-management and control.

## Methods

### Research design

The qualitative methodology of visual-based narrative enquiry was applied in this study using the Mmogo-method®. The Mmogo-method® is a context-sensitive approach that, through visual projection, could provide a deeper understanding of the meaning people attach to their interaction with others (Roos 2012). This method is fundamentally similar to focus groups; however, instead of the data depending mainly on group discussions, the participants construct their answers with indigenous material (Barbour 2013), such as malleable clay, straw and beads of different colours and shapes.

### Context of research

#### Diabetes peer support intervention

This study, which explored the experiences of peer support, was preceded by the Thaba ‘Nchu Botshabelo (TNB) Diabetes Peer Support Intervention. The purpose of the TNB Diabetes Peer Support Intervention was to establish the impact of the peer support intervention on adults with type 2 diabetes mellitus in terms of glycated haemoglobin (HbA1c), blood pressure, body mass index and waist circumference. In this 4-month, randomised control trial, individuals were eligible to participate if they were older than 18 years of age, had been diagnosed with type 2 diabetes, had no debilitating medical or related conditions, spoke Sesotho and were willing to participate. Community health workers (CHWs) from the purposively selected communities were trained as peer supporters, and they were each allocated to five individuals diagnosed with type 2 diabetes. Every month these individuals attended group sessions related to various aspects of diabetes, which were facilitated by the CHWs. Community health workers also conducted home visits every month to reinforce information and to provide peer support. The peer support training and intervention were based on the principles of motivational interviewing, which emphasise encouraging, guiding and non-judgemental support directed towards improving diabetes self-management. The clinical outcomes of the randomised control trial were reported separately in a doctoral thesis by the main author.

#### Setting

This study explored the experiences of peer support in two groups from the community of Thaba ‘Nchu in the Free State Province, South Africa. The majority of patients in this community make use of the public healthcare system and speak Sesotho. The researchers were not able to accurately determine the number of patients diagnosed with type 2 diabetes in the community of Thaba ‘Nchu.

#### Sampling method

Participants in the TNB Diabetes Peer Support Intervention were invited to take part in this study, which explored their experiences of peer support. Purposive sampling was carried out, and participants were selected based on their willingness to participate in the study. Data saturation was reached after two discussion groups, with a total of 12 participants. Table 1 represents the demographic data of participants. All the participants in the study were female; the age of participants ranged from 49 to 84 years. The participants had been living with diabetes for 1–21 years, and their education level varied. The majority of the patients had comorbidities, and the HbA1c of the groups ranged from 5.4% to 12.8%.

**TABLE 1:** Participant demographics (*N* = 12).

Characteristic	Group 1 (*n* = 6)	Group 2 (*n* = 6)
**Age, years**		
Range	51–84	49–69
Mean	65	60
**Duration of diabetes, years**		
Range	1–21	2–10
Mean	10	6
**HbA1c, %**		
Range	5.4–8.9	5.7–12.8
Mean	6.9	9.2
Gender, female (%)	100	100
Having comorbid conditions (%)	83	83
**Level of education (%)**		
Primary school	33	17
Some high school	67	0
Completed high school	0	83

### Data collection

The research team consisted of one English-speaking researcher, one Sesotho-speaking researcher and one Sesotho-speaking fieldworker, with the latter assisting with field notes. Data were collected at the primary healthcare facilities in an environment conducive to group discussions. Data were collected using the Mmogo-method®, which consists of the following four parts:

**Part 1:** All parties were introduced to each another, and the purpose of the study was explained to the participants by the Sesotho-speaking researcher. Although the participants could speak English, they had the option to speak in their native language, Sesotho.

**Part 2:** Participants were seated in a circle and asked the following question: ‘Can you please make a picture of how you experienced the group sessions and the home-visits during the peer support intervention’. Each participant was provided with malleable clay, beads of different colours and shapes, dried grass straw of various sizes and a round piece of cloth to build their visual constructions on. All participants completed their designs in approximately 45 min.

**Part 3:** Each participant was given an opportunity to describe his or her visual construction. Member checking occurred at this stage to verify the experiences of the participants. After the individual participants had given explanations, the group members could add to information shared by the participant.

**Part 4:** Participants were debriefed and, in the group, reflected on their experience of the activity. The researchers also debriefed immediately after the activity. The entire process was audio-taped, transcribed verbatim and translated to English – this served as textual data. Field notes were also made during the group discussions. Visual designs were photographed and presented as visual data.

### Data analysis

Triangulation of textual data from transcribed audio recordings of discussions, visual data from photographs of constructions and field notes was performed, and this led to themes that were analysed. The co-coder and researchers coded data independently, after which consensus discussion led to the emergence of themes that describe the experiences of participants during the diabetes peer support intervention. Direct quotes of participants were used to demonstrate and verify the emerging themes.

### Trustworthiness

The researcher applied Lincoln and Guba’s criteria of trustworthiness in the study (1985). The triangulation of textual and visual data during data analysis, as well as member checking during group discussions, contributed to *credibility. Transferability* was ensured by the provision of a detailed research context. The researcher, furthermore, engaged with participants until data saturation was reached. Verbatim quotes from participants in the findings of the study and photographs of visual presentations ensured *dependability. Confirmability* was achieved by the research assistant taking field notes during the group discussions, as well as the use of an independent co-coder during data analysis and consensus discussions.

### Ethical considerations

The Health Sciences Research Ethics Committee of the University of Free State (UFS-HSD2017/1546) provided ethical approval for the study, and the Free State Department of Health granted permission to conduct the study.

This study was guided by the ethical principles of beneficence, respect for people and justice in accordance with the Belmont Report (United States Department of Health and Human Services 1978). Participants provided written consent and permission to record the activities after an explanation regarding the aims, benefits and possible risks of the study. The participants were also informed that their participation was voluntary and that they could withdrew at any stage without penalty.

## Results

The following themes that relate to the experiences of the participants of the TNB Diabetes Peer Support Intervention emerged during data analysis: (1) positive lifestyle changes, (2) continuous support, and (3) improved confidence and a sense of connectedness with other participants. Direct quotes and visual constructions of the experiences of the diabetes peer support intervention are presented as evidence.

### Theme 1: Positive lifestyle changes

**FIGURE 1:**
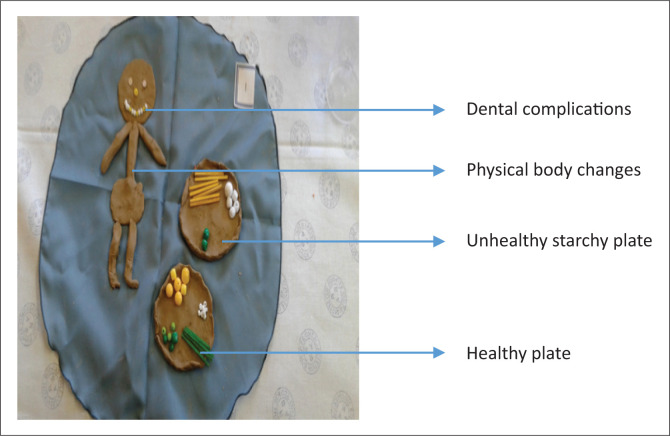
Positive lifestyle changes.

The participants in this study found that the TNB Diabetes Peer Support Intervention contributed to positive lifestyle changes, which were brought about by regular conversations on diabetes-related topics during group sessions and home visits. Participants started to have a better understanding of diabetes and the range of related physical changes and symptoms. Their understanding of symptoms related to prolonged high levels of blood glucose, which lead to complications of diabetes, also became clearer. They believed the information placed them in a position to make informed decisions, and consequently, the participants adjusted their diets and improved their activity levels. Participants articulated the following:

‘I have made two plates (Figure 1). First plate [shows the top plate] is for food that is not healthy; a big portion of maize meal pap, many chips with little vegetables, this is wrong. The second plate [*shows bottom plate*] is for healthy food! Portion of vegetables, small portion of pap and different food. I also have problems with my gums, some of my teeth falls out. The yellow here shows the lost teeth. Diabetes is a problem to eyes; it affects eyesight and my body lost weight as you see me.’ (Participant 1, group 1, 51 years old)

‘I designed this person, she is exercising, and she stretches her arms, doing the coconut exercises, which is the exercises she loves. Exercising makes the sugar go down. She likes vegetables and fruits, for example apples, carrots, cabbage, spinach, and potatoes.’ (Participant 6, group 2, 55 years old)

To support this theme, another participant said:

‘I was eating a lot, Sisters, but since we were taught about types of food, I reduce from eating a lot and I look for which food I can eat.’ (Participant 3, group 2, 69 years old)

Ironically, some participants did not readily accept weight loss because of lifestyle changes:

‘I want to comment on weight loss because of diabetes. I was wearing size 48. Since I lost weight, I have to wear a small size. I did feel bad about it, however, I have accepted it is a disease that is in me I know now where it comes from.’ (Participant 3, group 1, 51 years old)

‘From here I am slender, I am not in my normal body, you can see the head is big and heavy. The sugar makes my head gets dizzy and feels big when it is high.’ (Participant 2, group 1, 67 years old)

### Theme 2: Continuous support

**FIGURE 2:**
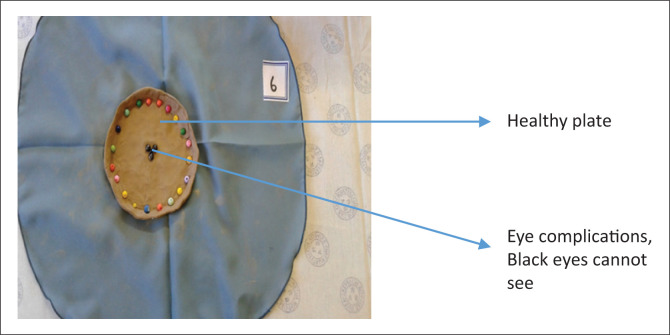
Continuous support.

The participants expressed that the continuous support offered by the CHWs was of great value to them – they also expressed an overwhelming sense of gratitude. They acknowledged that CHWs provided important support for them to continue with behavioural change, especially considering their day-to-day struggles with diabetes:

‘I made a plate of food. This plate is a plate of healthy food (Figure 2). I used to vomit after my supper I was not aware that I was eating a lot of food. After continuous discussions that we have to eat small portion of food, there was changes, the vomiting stopped.’ (Participant 6, group 1, 72 years old)

The blackness here represents when I was blind, I was referred to the eye clinic where they removed the layer that made me blind.’ (Participant 3, group 1, 72 years old)

Participants acknowledged the difficulty of dealing with diabetes, and therefore, they valued the interest the CHWs showed in them, as well as the time spent supporting them. Participants felt free to ask questions and express their fears and concerns to the CHWs in the study:

‘I would like to express my gratitude. I would say, all these things I never take them serious, until I attended the sessions every time and I learn the importance of them. I see now that they work.’ (Participant 4, group 2, 49 years old)

‘Yes Madam, I am very happy and thankful that I am more informed, as sugar is a daily problem. It is difficult to have sugar. Fruits are very much important and we were told every visit how to eat them.’ (Participant 4, group 1, 62 years old)

### Theme 3: Improved confidence and sense of connectedness

**FIGURE 3:**
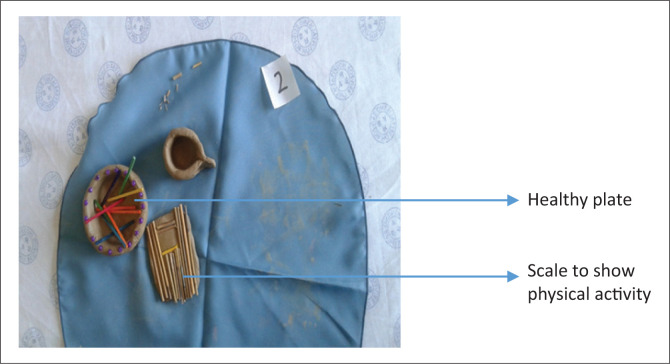
Improved confidence and sense of connectedness with others.

Participants reported that their confidence with regard to the self-management of diabetes increased over time. The confidence was brought about by improvements in physical health and self-reported clinical outcomes, such as blood glucose levels and blood pressure readings:

‘My life is different … it has changed. We were taught to eat fruits and vegetables [*see the bowl*] to control sugar (Figure 3). I am a person who prefer motogo (maize meal), it is always available day to day. I have come down with my portion size of motogo. I am losing weight [*see the scale*] by doing some training, things I never do, I do.’ (Participant 5, group 2, 60 years old)

‘You know, Mama, my sugar will push up and up and eventually I would be hospitalised. It could not be controlled. I was just trying the program to see if it will work and because I have been admitted in the hospital several times because of the sugar. That is why I am saying thank you Mama, thank you *[participant very emotional, tearful].* Since I started, I have not been admitted and the last time when I arrived here my ‘high blood pressure’ was also normal. I applied everything that we were taught here. I was curious about the adult school, not knowing how much it saved our lives.’ (Participant 6, group 2, 55 years old)

The confidence of the participants was a recurring observation in the field notes, as was the connection between the participants. The participants were very proud of their constructions and very confident when describing their way of doing things. The participants articulated the following:

‘We were taught many things. As I am one with diabetes, that is not controlled, it goes up. She taught us to drink water, to eat fruits and vegetables! As I speak, my sugar is better. I also know now, that others have sugar too. I am not alone.’ (Participant 2, group 2, 58 years old)

‘My eyes I made them to be clear I did not use a dark colour, My eyes and mouth are not dark anymore, they are bright because I am smart now because of what I know about diabetes.’ (Participant 6, group 2, 55 years old)

‘Here is a leg of a person with diabetes showing if you have diabetic ulcers on your foot or on your leg, you must consult your doctor. As a diabetic you do not stay with any wound on your body, as you do not know, it can get worse, so consult your doctor early.’ (Participant 4, group 2, 49 years old)

## Discussion

In this qualitative study, the experiences of adults who participated in the TNB Diabetes Peer Support Intervention, using the Mmogo-method®, were explored. The participants valued the intervention and acknowledged that the CHWs were an important source of support to them. Participants expressed that the intervention helped them to make positive lifestyle changes, and because they were exposed to the support continuously, their confidence in the self-management of diabetes improved. In addition, the intervention developed a sense of connectedness with other participants.

Positive lifestyle changes featured as an outcome of the TNB Diabetes Peer Support Intervention. A qualitative study conducted by Paul et al. (2013) in Ireland on the experiences of participants, peer supporters and practice nurses during a diabetic peer support intervention found similar positive effects of peer support. These participants attended nine peer support sessions presented by trained peer supporters who had type 2 diabetes themselves over a 2-year period. Each session covered specific aspects of diabetes. Participants in both this TNB Diabetes Peer Support Intervention and the Ireland study valued the support and comfort they received, new information that was picked up, lifestyle changes that were made and improvements in self-care. A study by Heisler and Piette (2005), conducted in the United States of America, evaluated the feasibility and acceptability of peer support by interactive voice response amongst older adults with diabetes. Patients with poorly controlled HbA1c >8% were paired with trained peer supporters who also had diabetes for a period of 6 weeks. Participants were matched based on their use of insulin or not and their diabetes goals or problems. Participants had to call their partner once a week on a toll-free interactive voice response line to provide support. The study showed that the support and assistance from their partners improved the participants’ motivation to follow healthy lifestyle, as well as their confidence regarding diabetes self-care. This is congruent with the results of the TNB Diabetes Peer Support Intervention, namely that gaining knowledge and taking responsibility can initiate a change in attitude and behaviour and may bring about positive lifestyle changes.

Continuous support featured as another important factor of peer support. The literature acknowledges the physically and emotionally demanding nature of diabetes self-management and the need for continuous support and motivation (Carpenter, DiChiacchio & Barker 2019; Warshaw et al. 2019). Expressions of support may include listening, acceptance (Peers for Progress 2015), showing how to do something (Simmons et al. 2015) or connecting the individual to a health, social or other community resources (Tsolekile et al. 2014). Yin et al. (2015) confirmed the significance of continuous support in peer support when they evaluated a peer support programme on metabolic and behavioural parameters in Chinese patients with type 2 diabetes. Trained peer supporters with type 2 diabetes provided telephonic support to the patients for 4 years. The support consisted of a telephone call of 15–20 min twice a week for the first 3 months, monthly for the second 3 months and every 2 months for the next 6 months. For the other 3 years, peer supporters were asked to contact their patients every 1–2 months. The peer supporters used a checklist to review medication adherence, diet, exercise and glucose monitoring. The authors found that after 4 years, the ongoing peer support had improved self-care behaviour, psychological health and glycaemic control of the participants. Standards of medical care in diabetes provided by authoritative bodies, such as the American Diabetes Association and the Society for Endocrinology, Metabolism and Diabetes of South Africa, include continuous monitoring and support of patients with diabetes to achieve their goals of diabetes self-management care, and this substantiates the findings of this study (American Diabetes Association 2019; SEMDSA Type 2 Diabetes Guidelines Expert Committee 2017).

Peer support improved the participants’ confidence in the self-management of diabetes and developed a sense of connectedness with other participants. A study conducted in the United States of America explored the perceptions of a peer support programme of veterans with type 2 diabetes. The veterans participated in a 6-month intervention that paired them with a trained peer supporter with type 2 diabetes who was also a veteran. The peer supporter called the veteran over phone once a week for 6 months to promote diabetes self-management. The authors observed that the veterans attached meaning to the intervention and felt connected to each other. Furthermore, they gained information through the interaction and gained confidence in their actions, because their blood glucose levels improved (Lott et al. 2019). Embuldeniya et al. (2013) conducted a qualitative analysis of 25 studies on the experience and impact of chronic disease peer support interventions; their study confirms the findings of the participants who underwent the TNB Diabetes Peer Support Intervention. Various concepts were associated with patient experiences of peer support, such as a sense of connection with each other; finding meaning in life; isolation prompting peer support; sharing of experiences; and a change in outlook, behaviour and knowledge, and empowerment. The evidence is similar to the findings of the TBN Diabetes Peer Support Intervention in terms of a change in knowledge and behaviour and a sense of connectedness with others. It is interesting to note that Embuldeniya et al. (2013) also highlighted the possibility of isolation occurring within peer support. This could refer to a situation of division existing between the patient and the peer supporter because of personality differences or different backgrounds.

An unexpected finding was the response to consequences of lifestyle changes reported by participants. The consequences of lifestyle changes, such as weight loss, may not have been readily accepted by the participants. In the African culture, a full body size may be associated with health, prosperity and beauty, and a slim body size may be associated with illness and disease (Micklesfield et al. 2013; Puoane, Tsolekile & Steyn 2010). Furthermore, obesity and overweight in women may be taken to reflect the husband’s ability to take care of his wife and family (Puoane et al. 2005); full-bodied women may even be treated with more respect and dignity (Puoane et al. 2005, 2010) because of their perceived ideal body size (Okop et al. 2016). Losing weight during the course of diabetes management made some of the participants feel inadequate, and they may even experience disapproval from the community. The evidence emphasises the significance of cultural influences, which have to be acknowledged and addressed, as it could create a possible barrier to the self-management of diabetes.

## Limitations

Despite all efforts to create an environment conducive to discussion groups, avoiding disruptions in the community setting remains challenging. During one discussion group, a moment of high noise levels in the healthcare facility may have disrupted the discussion groups. However, this disruption appeared to have a minimal impact on the participants as they carried on as if it was a norm.

It is unknown whether participants’ positive lifestyle changes, improved confidence and sense of connectedness with other participants would be sustained if a less structured peer support had been provided. There is concern whether the experience of positive lifestyle changes will be sustained over time, especially when the peer support intervention is no longer available. The generalisability of the study may be limited because of the specific context of the study. It is interesting to note that only positive experiences were identified during the study, in spite of measures taken to encourage participants to verbalise negative experiences.

## Recommendations

The findings of this study augment the current literature that confirms the positive effect of diabetes peer support interventions. These positive effects may lead to participant empowerment in terms of self-care, confidence, desire to change behaviour and, ultimately, improved diabetes self-management.

The study confirms that CHWs are well positioned in the health system to provide peer support to control and sustain diabetes self-management. Therefore, the healthcare system should provide clear guidelines to CHWs with regard to remuneration, training, supervision, support and monitoring.

Cultural perceptions of patients should also be taken into consideration during the development of diabetes peer support interventions.

## Conclusion

The study revealed that patients with type 2 diabetes from the community of Thaba Nchu in the Free State valued the TNB Diabetes Peer Support Intervention. The continuous support and interaction of the CHWs were experienced positively and brought about positive lifestyle changes, improved confidence and sense of connectedness with other participants. The influence of cultural perceptions related to obesity and overweight in the self-management of type 2 diabetes was enlightening.
